# Computer Simulation of the Electrical Stimulation of the Human Vestibular System: Effects of the Reactive Component of Impedance on Voltage Waveform and Nerve Selectivity

**DOI:** 10.1007/s10162-022-00868-w

**Published:** 2022-09-01

**Authors:** Simone D’Alessandro, Michael Handler, Rami Saba, Carolyn Garnham, Daniel Baumgarten

**Affiliations:** 1 Institute of Electrical and Biomedical Engineering, UMIT - Private University for Health Sciences, Medical Informatics and Technology, Hall in Tirol, Austria; 2 MED-EL GmbH, Innsbruck, Austria

**Keywords:** vestibular system, human anatomy, Fourier finite element method, dielectric tissues properties, electrode-tissue interface

## Abstract

The vestibular system is responsible for our sense of balance and spatial orientation. Recent studies have shown the possibility of partially restoring the function of this system using vestibular implants. Electrical modeling is a valuable tool in assisting the development of these implants by analyzing stimulation effects. However, previous modeling approaches of the vestibular system assumed quasi-static conditions. In this work, an extended modeling approach is presented that considers the reactive component of impedance and the electrode-tissue interface and their effects are investigated in a 3D human vestibular computer model. The Fourier finite element method was employed considering the frequency-dependent electrical properties of the different tissues. The electrode-tissue interface was integrated by an instrumental electrode model. A neuron model of myelinated fibers was employed to predict the nerve responses to the electrical stimulus. Morphological changes of the predicted voltage waveforms considering the dielectric tissue properties were found compared to quasi-static simulations, particularly during monopolar electrode configuration. Introducing the polarization capacitance and the scar tissue around the electrode in combination with a power limitation leads to a considerable current reduction applied through the active electrode and, consequently, to reduced voltage amplitudes of the stimulus waveforms. The reactive component of impedance resulted in better selectivity for the excitation of target nerves compared to the quasi-static simulation at the expense of slightly increased stimulus current amplitudes. We conclude that tissue permittivity and effects of the electrode-tissue interface should be considered to improve the accuracy of the simulations.

**Supplementary Information:**

The online version contains supplementary material available at 10.1007/s10162-022-00868-w.

## Introduction

**Fig. 1:**
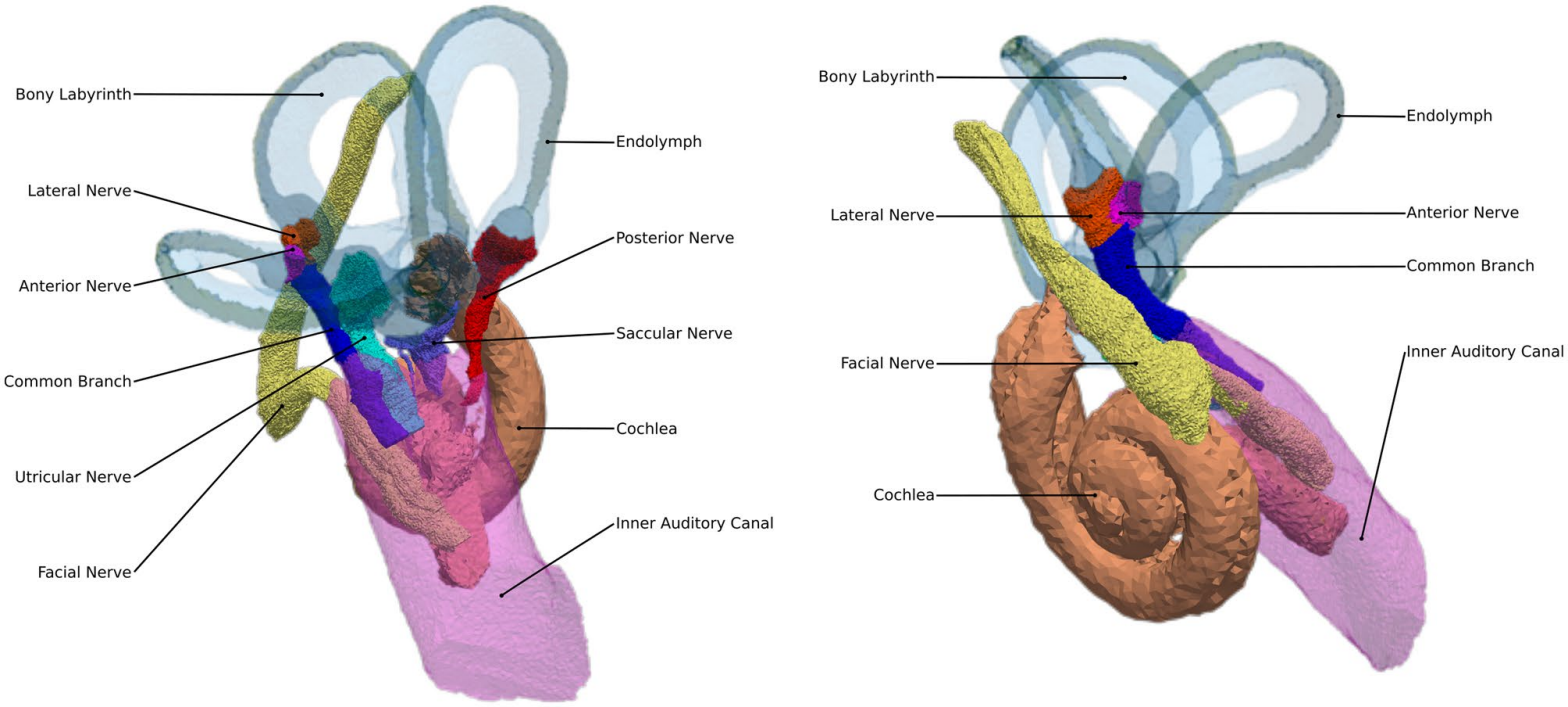
Labeled tetrahedral mesh of the human vestibular system anatomy used in our simulations from two different prospectives. The rotational accelerations are sensed by the nerves innervating the three SCCs (posterior, lateral and anterior), while the horizontal and vertical accelerations are sensed by utricle and saccule, respectively

The human inner ear is composed of two structures: the *cochlea*, responsible for hearing, and the *vestibular organ*, responsible for the sense of balance. Compared to vestibular dysfunction, hearing losses are relatively well understood, with several objective measures supporting differential diagnoses of auditory disorders [1]. The sense of balance and spatial orientation is strongly connected with the correct functionality of the vestibular system. It contributes to the stabilization of gaze during head motion through the vestibulo-ocular reflex and to postural control and spatial orientation by other pathways, such as proprioception [2]. The vestibular system is composed of three semicircular canals (SCC) and two otolith organs, utricle and saccule. Whereas the sensory epithelia in the ampullae of the SCC sense rotary motion, the hair cells on the sensory epithelia of the otolith organs perceive horizontal and vertical acceleration, respectively (see Fig. 1). Those structures are innervated from five nerve branches: posterior, anterior, lateral, utricular, and saccular nerve. The vestibular nerve (the combination of the superior and inferior divisions of the vestibular ganglion) combines with the cochlear nerve to form the vestibulocochlear nerve, which travels with the facial nerve through the internal auditory canal to the brain [3].

Typical symptoms of vestibular dysfunction are debilitating, e.g., with patients who suffer from vestibular loss facing embarrassment, permanent imbalance, difficulties walking, falls, blurred vision, concentration difficulties, memory impairment, disorientation in space, and even muscular pain [4]. Vestibular dysfunction can be caused by ototoxic effects, genetic abnormalities, Meniere’s disease, meningitis, ischemia, autoimmune disease and idiopathic or iatrogenic injury [5].

Vestibular implants offer a possible treatment option for patients with vestibular dysfunction. Accurate electrode positions and optimal stimulus waveforms are required for a selective stimulation of vestibular nerve fibers to partially restore vestibular sensation [6]. The feasibility and efficacy of vestibular implants have already been demonstrated in experiments on animals (e.g., [7–9]) and humans (e.g., [6, 10, 11]).

In recent years, several computer models of the vestibular system have been presented. Hayden et al. [12] realized a 3D model based on vestibular organs of chinchillas. Different electrode configurations, stimulus waveforms, and amplitudes were tested using spherical electrodes located inside the bony labyrinth. The extracellular potential field was computed considering the tissues to be purely conductive. Their results were validated by comparing the angular vestibulo-ocular reflex obtained by in vivo experiments with corresponding simulated results. Hedjoudje et al. [13] extended this model based on the rhesus temporal bone, and the same validation procedure was applied. On the other hand, Marianelli et al. [14], using an analogous approach, developed a simplified model based on human vestibular anatomy simulating the extra-labyrinthine approach. Moreover, Handler et al. [15] and Schier et al. [16] developed more complex computer models of the human inner ear considering electrodes placed at both intra- and extra-labyrinthine locations. Additionally, in their work the electrical neural model described by Hayden et al. [12] for chinchillas was adapted to the human vestibular anatomy to evaluate the nerve fiber activation.

The assumption of quasi-static conditions using purely resistive components without considering time and frequency-dependency of the tissue conductivity or reactance was employed in all the above-mentioned computer models. This simplification was claimed to be justified because dielectric relaxation times in cochlear tissues were shorter than the time scale of the applied stimuli, and 95 % of the spectral energy of the tested stimulus waveforms was below 12.5 kHz [13, 15, 17]. On the other hand, it has been shown in literature that the quasi-static approximation is only valid if [18]: $\omega \epsilon _0 \epsilon _r$/$\sigma$ $<<$ 1. This means that capacitive effects (angular frequency ($\omega$), vacuum permittivity ($\epsilon _0$) and relative permittivity ($\epsilon _r$)) must be considerably smaller compared to the conductivity ($\sigma$) to be negligible in simulations. This is not valid for all the tissue structures in the 3D computer model. Moreover, experiments showed that the reactive component of impedance can affect the shape and amplitude of the voltage waveform [19]. Furthermore, the electrode-tissue interface and the scar tissue, developing around the electrodes after implantation [20], as well as their effects on stimulation outcome and power consumption, have not been considered in computer simulations of vestibular implants.

In this work, the effects of the reactive component of impedance, the electrode-tissue interface, and the scar tissue and corresponding nerve fiber activation are investigated for the first time in a realistic 3D model of a human vestibular system. The influence of the reactive component of impedance is investigated by considering the frequency-dependent admittivities of the different structures applying the Fourier finite element method (Fourier FEM). The frequency-dependent dielectric properties considered during the simulations are based on values found in literature [21, 22] and are characterized by uncertainty, since the exact values are still unknown. For this reason, specific ranges within which the tissue properties may vary are defined and analyzed. Furthermore, an instrumental electrode model was implemented in order to consider the electrode-tissue interface and the scar tissue surrounding the electrodes. In addition, a power limitation is considered to properly evaluate these effects.

## Methods

### Overview

According to Inguva et al. [18] the quasi-static approximation (QS) previously employed in other models is only valid if: $\omega \epsilon _0 \epsilon _r$/$\sigma$ $<<$ 1. By considering the tissue properties of the different structures (more details in "Tissue Properties") the above-mentioned QS criterion has been calculated for some frequency components (see Table 1). As can be seen, this ratio is considerably lower than 1 only for the cerebrospinal fluid, while for the other structures this assumption is not valid for all frequencies. Moreover, in one of our recent works [23] we measured the impedance in the vestibular system of a guinea pig between 100 Hz and 50 kHz and we compared the measurements with a lumped parameter model. To get a proper agreement with the measurements, the lumped parameter model was described by a combination of resistors and capacitances. It was shown that the reactive component of impedance needs to be considered to increase the agreement between measurement and simulation.

**Table 1:** The quasi-static criterion at five frequency components obtained from the DFT of the long stimulus waveform for the different tissue structures considered in the simulation. Nerve* summarizes longitudinal and transversal directionality of the dielectric properties of the nerves

Material	$\omega \epsilon _0 \epsilon _r$ / $\sigma$				
	f = 2 kHz	f = 10 kHz	f = 100 kHz	f = 150 kHz	f = 200 kHz
Bone	0.0092	0.0139	0.0628	0.0875	0.1106
Nerve*	0.2084	0.4634	0.3462	0.3149	0.2996
CSF	6.0707e-6	3.0354e-5	0.0003	0.0004	0.0006

A computer model was created with the aim of evaluating the voltage waveforms caused by simulated electrical stimulation of vestibular target nerves and analyzing vestibular nerve fiber activation. The 3D model considered in this work is based on µCT scans and consists of the vestibular system (endolymphatic and perilymphatic space, posterior nerve, anterior nerve, lateral nerve, utricular nerve, saccular nerve), the cochlea (cochlear nerve, scala tympani, scala vestibuli, and scala media), the facial nerve, and the inner auditory canal (IAC; see Fig. 1). The region surrounding the vestibular system was neither segmented nor directly considered in the model because it was incomplete after extraction of the specimen. For this reason, the surrounding structures were modeled as homogeneous domain consisting of bone tissue surrounded by a saline layer. A more detailed description of the model is given in "Model Consideration and Stimulus Waveforms". Based on this model, the reactive component of impedance has been considered by using the Fourier FEM approach, in which the potential distribution is evaluated for various frequency components of the stimulus waveform considering frequency-dependent tissue admittivities. The dielectric properties for the different tissues have been obtained from literature, and respective ranges have been defined [12, 21, 22, 24]. Furthermore, an instrumental electrode model is applied to consider a more realistic behavior of the electrodes and considered in the simulation environment in combination with the power limitation aspect. The voltage waveforms are used as extracellular potentials in the applied neural models for the evaluation of selective nerve fiber activation. The effects are analyzed separately: first, effects arising from the consideration of frequency-dependent admittivities of vestibular tissues on the voltage waveforms are evaluated. Subsequently, the electrode-tissue interface is considered in the simulations, followed by an analysis of the impact of scar tissue layers surrounding the electrodes. Finally, recruitment curves for nerve fiber activation are simulated using the time-dependent potential distributions obtained by the different stimulation scenarios as input for the neural model.

### Fourier Finite Element Method

The Fourier FEM was implemented to predict the effects of the reactive component of impedance. The Fourier FEM can be summarized in the following steps [19]: first, the stimulus waveform to be applied through the electrode contact is constructed in the time domain. In the next step, the stimulus waveform is converted from the time domain to the frequency domain using a discrete Fourier transform (DFT), which provides the magnitude and phase that constitutes the stimulus waveform. For each of the frequency components, the Laplace equation is solved (see below) considering frequency-dependent tissue admittivities and capacitive components introduced by the electrode-tissue interface, yielding a complex valued potential distribution. The predicted potential distribution in the frequency domain is converted back to the time domain at specific points in the model by applying the inverse discrete Fourier transform (IDFT).

### Partial Differential Equations

Under consideration of QS assumptions (purely conductive tissue properties without frequency-dependency), potential distributions caused by electrical stimulation in tissue can be calculated by the Poisson equation [14]

1 $$\begin{aligned} -\nabla \cdot [\varvec{\sigma }(\varvec{x})\nabla \phi (\varvec{x})] = \nabla \cdot \varvec{J}(\varvec{x}) = I(\varvec{x})\text {,} \end{aligned}$$

where $\varvec{\sigma }$ is the electrical conductivity (Sm^-1^), $\phi$ is the potential (V), $\varvec{J}$ is the current density (Am^-2^), and *I* is the current source density (Am^-3^) at point $\varvec{x}$ in the model.

In the absence of current source density in the model (the stimulation current is applied via a boundary condition, see "Boundary Conditions"), Eq. 1 becomes

2 $$\begin{aligned} -\nabla \cdot [\varvec{\sigma }(\varvec{x})\nabla \phi (\varvec{x})] = 0\text {.} \end{aligned}$$

By only using a real-valued electrical conductivity $\varvec{\sigma }$ in Eq. 2, the reactive component of impedance is not considered. To cope with this limitation, a frequency-dependent admittivity has been introduced. The electric potential distribution caused by a current stimulation (applied through the active electrode) was calculated by solving the Laplace equation with a complex admittivity at each frequency component obtained from the DFT:

3 $$\begin{aligned} -\nabla \cdot [\varvec{\gamma }(\varvec{x},f_i)\nabla \phi (\varvec{x},f_i)] = 0 \end{aligned}$$

4 $$\begin{aligned} \varvec{\gamma }(\varvec{x},f_i) = \varvec{\sigma }(\varvec{x},f_i) + j2\pi f_i \varepsilon _0 \varvec{\varepsilon _r}(\varvec{x},f_i)\text {,} \end{aligned}$$

where $f_i$ is the frequency component considered, $\varvec{\sigma }$ defines the electrical conductivity (Sm^-1^), $\varepsilon _0$ the vacuum permittivity (8.854 ×10^-12^), $\varvec{\varepsilon _r}$ the relative permittivity and *j* = $\sqrt{-1}$ is the imaginary unit. The frequency-dependent admittivity $\varvec{\gamma }$ (Sm^-1^) is described by a complex tensor, whereas the potential $\phi$ (V) is a complex scalar field.

### Boundary Conditions

**Fig. 2:**
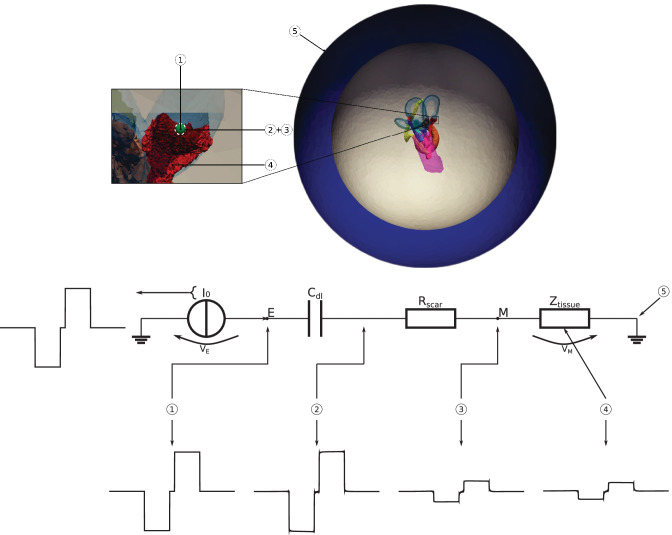
Representation of the 3D model between the active electrode and the reference electrode during monopolar stimulation scenarios. Top: Inner ear mesh embedded in bone sphere (gray) and saline layer (blue) used during the simulation to summarize the surrounding structures and a zoom-in at the active electrode position (located in the center of the ampulla of the posterior SCC, illustrated in green). The double-layer capacitance and the scar tissue are indicated with a white dashed line around the active electrode. Center: Long stimulus current waveform and the electrical equivalent circuit, schematizing the 3D model, considering the double-layer capacitance (C_dl_), the scar tissue resistance (R_scar_) and the overall tissues impedance (Z_tissue_). Bottom: Resulting voltage waveforms at the numbered positions in the 3D model. 1 is the position inside the active electrode; 2 is the location after C_dl_; 3 is considered after R_scar_; 4 is a location at the target nerve inside Z_tissue_; 5 is the voltage reference (0 V)

A particular boundary condition was implemented to consider more realistic behavior of the electrode and electrode-tissue interface. The instrumental electrode model introduced by Zhang and Li [25] allows modeling the contact impedance at the electrode surface together with capacitances and leakage currents of the cable and electrode. In the simulations described in this work, only the contact impedance of the electrode-tissue interface was considered from the instrumental electrode model, as this work focuses on effects related to tissue reactance. Figure 2 illustrates the 3D vestibular system surrounded by two concentric spheres (bone and saline layer) together with the equivalent electrical circuit between active and reference electrodes considering the electrode-tissue interface. The active electrode is considered as the current source in the performed simulations. The whole model can be represented as a current-controlled electrical circuit composed of a current generator (active electrode) and impedances connected in series between active and reference electrodes (see Fig. 2). The electrode-tissue interface is considered as an impedance (Z_ET_ = R_scar_ + 1/(j$\omega$C_dl_)), which is connected in series to the total tissue impedance (Z_tissue_). Moreover, Fig. 2 depicts exemplary voltage waveform results at the different positions in the 3D model.

During the simulation, the contact impedance of the electrode-tissue interface has been considered as the sum of two effects: polarization capacitance and resistance of surrounding scar tissue. The polarization capacitance was introduced in the simulation considering a double-layer capacitance (C_dl_) around the electrode. For a metal in an aqueous solution, the double-layer capacitance falls within a range of 10–20 µF cm^-2^ [26]. A mean value of 15 µF cm^-2^ was chosen and multiplied by the electrode surface area to obtain C_dl_. The resulting capacity was applied as reactance in the instrumental electrode model at each frequency component obtained from DFT. Moreover, the effect of the scar tissue impedance was defined as a homogeneous spherical structure around the electrode with a constant conductivity of 0.1 Sm^-1^ [27] and considered in the simulation as resistance in the instrumental electrode model. Various scar tissue thicknesses around the electrode were simulated: 300 µm, 400 µm and 500 µm. Only the results obtained considering 500 µm thickness are shown. The other simulations yielded expected behavior: the smaller the scar tissue is, the higher is the nerve fiber activation and vice versa.

The stimulus current applied via the active electrode contains the magnitude and the phase shift for each frequency component obtained by the DFT and was applied over a model boundary describing the surface of the electrode (see [25], for further details).

Different electrode configurations were considered in the model: in a *monopolar stimulation scenario*, the stimulus current is applied via a single electrode closely positioned to the targeted nerve, while in a *bipolar electrode configuration* a pair of electrodes closely located to the targeted nerve operates as a current source and current sink, respectively, with inverted current amplitudes. In both monopolar and bipolar electrode configurations, a reference electrode was considered by applying an instrumental electrode model at the outer surface of the model considering a reference voltage of 0 V.

### Tissue Properties

Frequency-dependent dielectric properties of biological tissues considered in the model are required for obtaining electrical potential distributions caused by the stimuli applied through the electrodes. Permittivity and conductivity for a wide frequency spectrum (from 10 Hz to 20 GHz) for several biological tissues were described by Gabriel et al. [22], using a model simulating the Cole-Cole equation to describe the values for the entire frequency range. This model is based on measurements performed by the group and values from the literature. From the work of Gabriel et al. [22], the values for conductivity ($\sigma$(f)) and permittivity ($\varepsilon$(f)) at different frequencies were extracted (thereafter called “default values”). Based on these properties, the frequency-dependent admittivity $\gamma$(f) can be defined as described in Eq. 4.

Regarding the tissue properties of the bone region surrounding the inner ear, values of conductivity ($\sigma$_bone_(f)) and permittivity ($\varepsilon$_bone_(f)) at different frequencies were obtained from the plot of the dielectric properties of the cortical bone.

Concerning the target nerve structures, the tissue properties of the spinal cord were chosen. Dielectric properties of neural tissue exhibit strong directional dependence: the electrical conductivity parallel to the nerve fiber orientation (*longitudinal*) is considerably higher compared to a direction orthogonal to the nerve fiber orientation (*transversal*). The dielectric properties of neural tissue summarized in the work of Gabriel et al. [22] are not related to a particular fiber orientation during measurement. To consider the anisotropy inside the nerve, the conductivity at a low frequency (100 Hz) with the default value ($\sigma$ = 0.0295 Sm^-1^) was used as reference value, which was scaled according to the longitudinal and transversal conductivity values given by Hayden et al. [12]. Using this approach, frequency-dependent admittivities of neural tissue for longitudinal direction ($\gamma _{\text {nerve,l}}$(f)) and transversal direction ($\gamma _{\text {nerve,t}}$(f)) were obtained. The same procedure was used for defining the electrical properties of the cochlear nerve, but no anisotropy was considered for this nerve, as the level of detail in the anatomical model was insufficient to generate nerve fibers in the fine structures of the cochlear nerve. The frequency-dependent electrical conductivity and permittivity of the spinal cord described by Gabriel et al. [22] were scaled to match the average longitudinal and transversal conductivity given by Hayden et al. [12] at 100 Hz to obtain a frequency-dependent admittivity $\gamma$_cochlea_(f) for the volume in the model representing the cochlear nerve. According to Finley et al. [28] the conductivity of the endolymph and perilymph are slightly different (1.67 Sm^-1^ and 1.43 Sm^-1^, respectively). Following the same simplification indicated in other computer models (e.g., [12, 14–16]) and in the absence of more precise tissue properties, the endolymph and perilymph are considered as one structure with the same dielectric properties. For this reason, tissue properties of cerebrospinal fluid (CSF), i.e., conductivities and permittivities ($\gamma _{\text {CSF}}$(f)), were used for the endolymph and perilymph within the bony labyrinth (consisting of the SCC, the vestibulum of the vestibular system, and the cochlea) as well as in the saline layer, since the perilymph is a derivative of CSF [24].

For investigating the influence of the tissue properties on the voltage waveforms, a specific range has been defined for each tissue within which the properties may vary. Considering the bone structure, this range was defined based on the literature values extracted from Gabriel’s plot. The logarithmic distance between the default values and the literature values was calculated for the different frequencies. The median value of all logarithmic distances was used to define this range, considering conductivity and permittivity separately. To define the lower ($\gamma _{\text {nerve,l}}{}^\downarrow$(f), $\gamma _{\text {nerve,t}}{}^\downarrow$(f), and $\gamma _{\text {cochlea}}{}^\downarrow$(f)) and upper ($\gamma _{\text {nerve,l}}{}^\uparrow$(f), $\gamma _{\text {nerve,t}}{}^\uparrow$(f), and $\gamma _{\text {cochlea}}{}^\uparrow$(f)) boundaries for dielectric properties of longitudinal and transversal nerve directionality and cochlea nerve, ±50 % of conductivity and permittivity at different frequencies were considered. Considering CSF, Baumann et al. [21] measured the electrical conductivity of human CSF at different frequencies, at body temperature. Those values were used to define the upper ($\gamma _{\text {CSF}}{}^\uparrow$(f)) and lower ($\gamma _{\text {CSF}}{}^\downarrow$(f)) boundaries, following the same approach described for the bone region. For a detailed view of the dielectric properties of the tissues and their range boundaries, the reader is referred to the Supplemental Figures.

During the simulation, each property was obtained by cubic spline data interpolation between the default values at certain frequencies and the frequency components considered during the simulation obtained from DFT. Where it is not specified, default values for all the tissue properties were used.

To evaluate the influence of varying the material properties in the simulation using the same anatomical model and same monopolar electrode configuration, the root mean square error (RMSE) of the predicted voltage waveforms was obtained at different positions in the model:

5 $$\begin{aligned} RMSE = \sqrt{\dfrac{1}{N}\sum _{i=1}^{N}(V_{i}^{A} - V_{i}^{B})^2} \end{aligned}$$

$V_{i}$ is the voltage at time instance *i* and *N* is the number of time steps. The superscripts *A* and *B* indicate the consideration of the default tissue properties and dielectric properties considered at the upper and lower boundaries, respectively. The time instance considered to calculate the RMSE for each waveform was taken from 40 µs before the onset of the stimulus to 40 µs after the stimulus ends.

### Power Limitation

Due to the serial connection of the components in the equivalent circuit (cf. Fig. 2), the same current (I_0_) flows through the two impedances (Z_ET_ and Z_tissue_). Therefore, the voltage (V_M_) present at the surface of the scar tissue (between Z_ET_ and Z_tissue_) is not influenced by the impedance caused by the electrode-tissue interface (Z_ET_). However, the potential of the active electrode (V_E_) is considerably increased when the scar tissue is considered, leading to an increase in power consumption. Due to safety limitations, the power applied during a stimulus is limited. Therefore, a power limitation was implemented to correctly evaluate the effects of the electrode-tissue interface.

A maximum power of 5 mW for electrical stimulation by a cochlear implant was described in the work of Saba [29]. The same value was also used as a power limitation for the simulated vestibular implant (P_MAX_ = 5 mW).

The power required at every time step is calculated by multiplying the voltage at the electrode by the current emitted by the electrode itself (P = I_0_V_E_), while the overall impedance between the active and reference electrode is obtained by dividing the voltage and current waveforms (Z_tot_ = Z_ET_ + Z_tissue_ = V_E_/I_0_). When the power (P) required at a time step was larger than the maximum power (P_MAX_), a new current was applied considering the power limitation (I_NEW_ = $\sqrt{\text {P}_{\text {MAX}}/{\text {Z}}_{\text {tot}}}$). This new current was then used to scale the predicted voltage waveform at each relevant point within the model.

### Neural Model

For each nerve branch in the model (except for the cochlear nerve), 400 nerve fibers were generated. The nerve fibers were distinguished into three different fiber types in relation to their position: *calyx afferent* (largely innervating the central part of the sensory epithelia), *bouton afferent* (principally innervating the peripheral region of the sensory epithelia), and *dimorphic afferent* (innervating the whole surface of the sensory epithelia). Each nerve fiber type yields different properties such as axonal diameter and frequency of occurrence (see [16], for further details).

An electrical neural model of human vestibular anatomy was implemented to estimate nerve fiber activation in different stimulation scenarios and to evaluate the selectivity of specific stimulus waveforms. For this purpose, the model developed by Schier et al. [16] was extended to consider potential distributions provided by the Fourier FEM and power limitation aspects. The electrical neural model is based on the *spatially extended nonlinear node* (SENN) model described by Hayden et al. [12] for chinchilla vestibular afferents and was adapted by Schier et al. [16] to the human vestibular system by changing the neuron morphology.

In the SENN model, each node of Ranvier is considered as an active node and schematized as two nonlinear channels (voltage-gated Na$^+$ and K$^+$ channels), a leakage channel, and a membrane capacitance. Two consecutive nodes of Ranvier in the same neuron are linked via axoplasmic conductance. The potential distribution obtained by solving the partial differential equation with a complex admittivity in the frequency domain was linearly interpolated at each node of Ranvier, converted back to the time domain via IDFT, and used as input for the neural model as extracellular potential.

The simulation was performed by using a binary search algorithm to properly scale the stimulus amplitude to determine the excitation threshold for each nerve fiber. The fiber was considered activated when the Na$^+$ channel activation parameter *m* was higher than 0.7 [30] and the binary search was terminated when the difference between the upper and the lower boundary was less than 0.1 %.

**Fig. 3:**
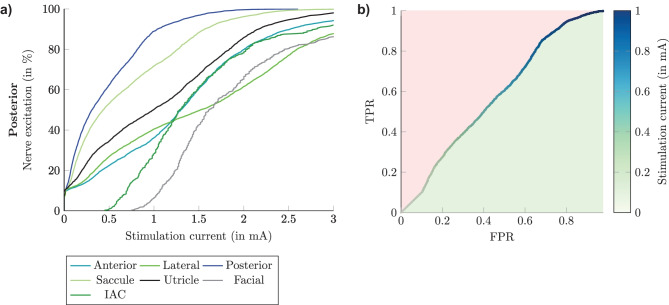
Exemplary fiber recruitment curves when applying the long stimulus waveform during monopolar electrode stimulation when the active electrode is located in the center of the ampulla of the posterior SCC and the corresponding ROC curve are shown in (**a**) and (**b**), respectively. The numerical integration of the green space in (**b**) is the AUC value considered for the selectivity in the worst-case scenario

To evaluate the selectivity of the neural stimulation, several approaches were already published [16, 31, 32] comparing the target nerve excitation curve with the mean of all non-target nerve activation curves or with a single activated non-target nerve (worst-case scenario). Receiver operating characteristic (ROC) curves were obtained by plotting the true positive rate (TPR) of the nerve activation against the false positive rate (FPR) by progressively increasing the stimulation current amplitude (see Fig. 3). The TPR and FPRs are the percentage of active nerve fibers in the targeted nerve and non-targeted nerves, respectively. The highest FPR is used to evaluate the selectivity of the worst-case scenario. The numerical integration of the ROC curve (area under the curve, AUC) is used to evaluate the selectivity of a stimulation. The AUC value is ranging between 0 and 1, where 0 means no activation of the target nerve and 1 implies optimal selectivity [16].

### Model Consideration and Stimulus Waveforms

Labeled 3D images based on high-resolution µCT recordings of a human inner ear, provided by the Medical University of Innsbruck, were used to generate a 3D tetrahedral mesh using a semi-automatic modular workflow [15], which involved TetGen [33] and CGAL [34] as meshing tools.

The model considered in this work is based on a vestibular specimen excised of a donated body of a 78-year-old male. This corresponds to *Model 3* in the work of Handler et al. [15].

The labeled components are embedded in a bone sphere with a radius of 25 mm to consider the temporal bone surrounding the vestibular system, which is further surrounded by a saline layer of 10 mm thickness to consider additional structures in the vicinity as previously described in the works of Handler et al. [15] and Schier et al. [16] and proposed by Marianelli et al. [14] (see Fig. 2). Subsequently, the nerve fiber orientation was modeled separately for each nerve branch to consider the anisotropic electrical conductivity and permittivity of the neural tissues in the model.

**Fig. 4:**
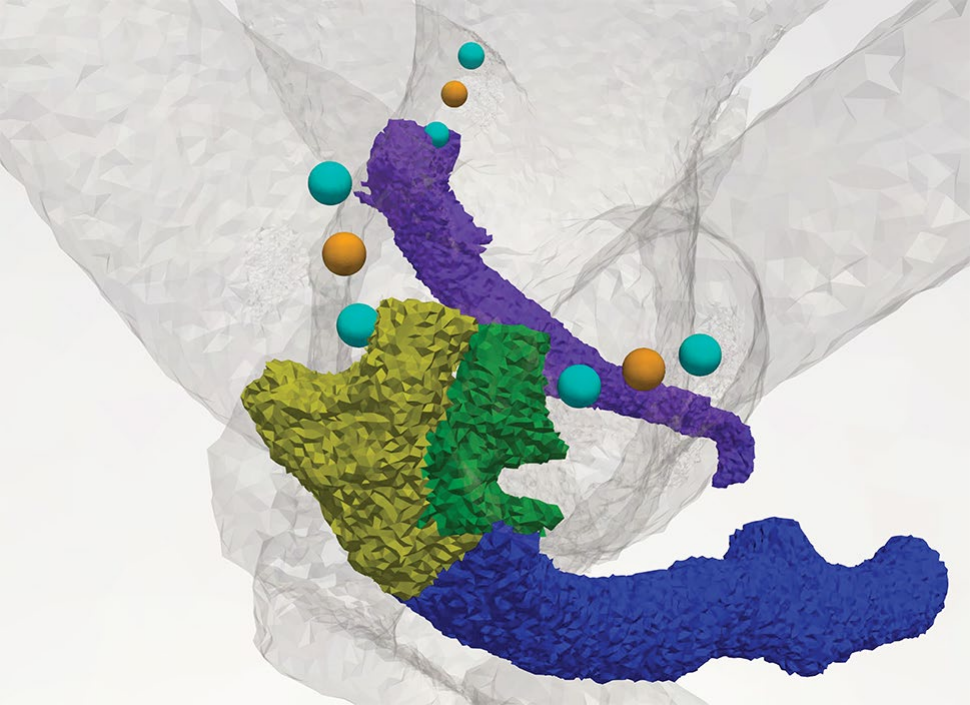
Electrode positions during monopolar (orange) and bipolar (light blue) electrode configurations for posterior (purple), anterior (green) and lateral (dark yellow) nerves. The common branch of the anterior and lateral nerves is depicted in blue

The electrode inserted into the model has a spherical shape with a radius of 150 µm. In the case of a monopolar scenario, the center of the active electrode is located 750 µm from the center of the sensory epithelia of the ampullary nerves along their transversal axis. Regarding bipolar electrode scenarios, transversal parallel electrode configurations were simulated: electrodes are located at distances of 250 µm and 1250 µm from the centers of the sensory epithelia of the posterior, anterior, and lateral nerves along the transversal axis (see Fig. 4). The volumetric mesh for the considered dataset had approximately 12 million elements.

**Fig. 5:**
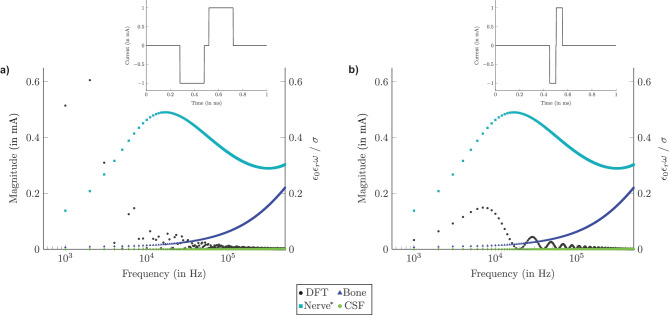
DFT overlapped with the QS criterion calculated for every structure with a frequency resolution of 1 kHz shown in a) and b) for long and short cathodic-phase-first charge-balanced biphasic current waveforms applied through the active electrode in time domain illustrated in the inlet, respectively. Nerve* summarizes longitudinal and transversal directionality of the dielectric properties of the vestibular nerves

Two cathodic-phase-first, charge-balanced biphasic current waveforms were used to perform the simulations and applied through the active electrode: a long stimulus waveform with a phase duration of 200 µs and a phase gap of 40 µs and a short stimulus waveform with a phase duration of 50 µs and a phase gap of 2 µs. Both stimulus waveforms have a stimulus amplitude of 1 mA (see inlet of Fig. 5a and b). These waveforms are standard waveforms with a negative phase first, as this would most likely directly activate the axon near the active electrode [35]. Both stimuli were converted to the frequency domain by performing a DFT in Octave [36] with a frequency resolution of 1 kHz and a maximum frequency of 500 kHz (see black points in Fig. 5a and b). This frequency resolution has been chosen as a good combination between computational time and signal reconstruction. For each frequency present in each spectrum, the magnitude and phase shift were considered as input during the Fourier FEM, separately. At every frequency component, also the dielectric properties for the different structures inside the model were accordingly assigned as mentioned in "Tissue Properties".

In addition to the magnitude of the DFT for both stimulus waveforms also the QS criterion for every structure at each frequency component is shown in Fig. 5a and b. The figure shows that the ratio between susceptivity ($\omega \epsilon _0 \epsilon _r$) and conductivity ($\sigma$) for nerve tissue is not considerably lower than 1 for frequency components with high magnitudes, similarly as shown in the work of Inguva et al. [18]. For shorter pulse widths, the frequency components with the highest magnitudes are shifted towards higher frequencies (see Fig. 5b), leading to a stronger capacitive effect due to higher tissue susceptivities.

The simulations were performed using in house developed C++ code also applied in Handler et al. [15] and Schier et al. [16], which was adapted to allow for solving the linear equation system by the MUltifrontal Massively Parallel sparse direct Solver (MUMPS, [37]) to be able to parallelize the solving process of the Fourier FEM and reducing the computational time to approximately 10 min for each frequency component.

## Results

### Predicted Voltage Waveforms

**Fig. 6:**
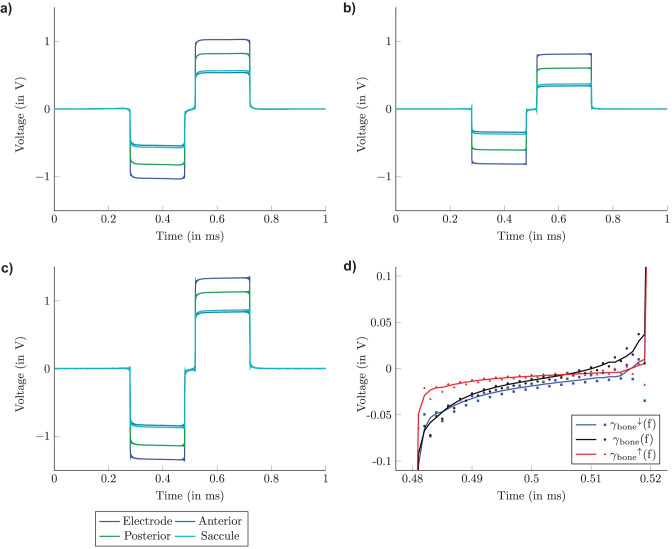
Predicted voltage waveforms obtained applying the long stimulus waveform at the active electrode and central points of the sensory epithelia of the posterior, anterior and saccular nerve by performing the Fourier FEM with monopolar electrode configuration in the ampulla of the posterior SCC changing the tissue properties of the bone structure: **a)** on $\gamma _{\text {bone}}$(f), **b)** on $\gamma _{\text {bone}}{}^\uparrow$(f) and **c)** on $\gamma _{\text {bone}}{}^\downarrow$(f). **d)** Comparison of the phase gap of the predicted voltage waveforms at the posterior nerve position when the tissue properties of the bone are considered at different boundaries. The depicted points are the outcomes of the simulations and the smoothed lines are obtained by applying a median filter (with a window of 10 µs) to emphasize the behavior in the phase gap for the three simulations

Predicted voltage waveforms obtained by considering the dielectric properties of the different tissues, applying the long stimulus waveform, and performing the Fourier FEM are presented in Fig. 6a. It shows the predicted voltage waveforms obtained at the surface of the active electrode and central points of the sensory epithelia of the posterior, anterior, and saccular nerves during monopolar electrode configuration when the active electrode was placed in the ampulla of the posterior SCC. The effects caused by also considering tissue permittivities in the model are clearly visible in the predictions. Whereas the inclination of the slopes between the baseline of the predicted voltage waveform and its maximum and minimum are equivalent in simulations without considering the reactive component of impedance (QS simulation), the slopes of the predicted voltage waveforms are decreased when considering the capacitive effects introduced into the model. Moreover, during the Fourier FEM, the edges of the predicted voltage waveforms are not as sharp compared to the QS simulation.

The effects obtained on the predicted voltage waveforms by changing the material properties of the bone on the upper ($\gamma_{\text {bone}}{}^\uparrow$(f)) and lower ($\gamma _{\text {bone}}{}^\downarrow$(f)) range boundary separately in different simulations during the same monopolar electrode configuration are shown in Fig. 6b and c, respectively. Therefore, considering the dielectric properties of the tissues, the predicted waveforms do not show a flat plateau at 0 V but rather a smooth transition from the cathodic to the anodic phase.

Referring to the simulation where $\gamma _{\text {bone}}{}^\uparrow$(f) is considered (Fig. 6b), the amplitude and the slopes of the predicted voltage waveforms are decreased compared to simulations considering default values or $\gamma _{\text {bone}}{}^\downarrow$(f). Moreover, before the stimulus appears (t = 280 ms) there is a spike on the voltage waveform due to the time resolution used to discretize the signal. This effect is present on all the edges of the curves: it is more pronounced at the position of the saccular nerve compared to the other considered locations. The same behavior can also be seen when $\gamma _{\text {bone}}{}^\downarrow$(f) is used. In this case, the spikes are visible for each considered point inside the mesh. Additionally, the slopes are increased again with respect to considering the $\gamma _{\text {bone}}{}^\uparrow$(f). The shapes of the phase gap of the voltage waveform at the central point of the sensory epithelia of the posterior nerve are depicted in Fig. 6d when default values ($\gamma$(f)), $\gamma _{\text {bone}}{}^\uparrow$(f), and $\gamma _{\text {bone}}{}^\downarrow$(f) are considered. The depicted points are the outcomes of the Fourier FEM, and the smoothed lines were obtained by applying a median filter (with a window of 10 µs) to emphasize the behavior in the phase gap. Using $\gamma _{\text {bone}}{}^\uparrow$(f), the slopes decreased compared to the other two curves. Moreover, the behavior when using the default values and $\gamma _{\text {bone}}{}^\downarrow$(f) is almost the same within 0.48-0.49 ms, but when considering the default values the waveform approaches its maximum more rapidly compared to $\gamma _{\text {bone}}{}^\uparrow$(f) and $\gamma _{\text {bone}}{}^\downarrow$(f).

To evaluate how the tissue properties influence the voltage waveforms, the RMSE was calculated and the maximum RMSE was obtained when $\gamma _{\text {bone}}{}^\downarrow$(f) was considered. In contrast, by considering $\gamma _{\text {CSF}}{}^\downarrow$(f) and $\gamma _{\text {CSF}}{}^\uparrow$(f), it can be noted how the voltage at the electrode is influenced by these tissue properties while the voltages at the other points show considerably smaller RMSE values. On the other hand, varying the tissue properties of the nerves gave almost the same RMSE for each point. The reader is referred to the Supplemental Figures for a 3D visualization of the RMSE values of the voltage waveforms obtained considering the same posterior monopolar electrode configuration comparing the default values of the dielectric properties with all the upper ($\gamma _{\text {bone}}{}^\uparrow$(f), $\gamma _{\text {nerve,l}}{}^\uparrow$(f), $\gamma _{\text {nerve,t}}{}^\uparrow$(f), $\gamma _{\text {CSF}}{}^\uparrow$(f)) and lower ($\gamma _{\text {bone}}{}^\downarrow$(f), $\gamma _{\text {nerve,l}}{}^\downarrow$(f), $\gamma _{\text {nerve,t}}{}^\downarrow$(f), $\gamma _{\text {CSF}}{}^\downarrow$(f)) range boundaries, respectively.

### Electrode-Tissue Interface and Power Limitation

Monopolar and bipolar stimulation scenarios were simulated considering a double-layer capacitance (C_dl_) of 15 µF cm^-2^ at the active electrodes (and also at the sink electrodes during bipolar electrode stimulation scenarios). While similar predicted voltage waveforms and nerve fiber activation were obtained during simulation of monopolar stimulation scenarios, effects of C_dl_ were visible at the electrode and target nerve for bipolar stimulation scenarios (predictions not shown).

**Fig. 7:**
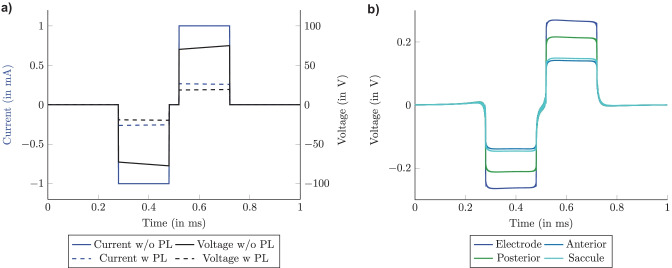
Influence of power limitation for long stimulus waveform: **a)** current and voltage waveforms at the active electrode without (solid lines) and with (dashed lines) power limitation; **b)** resulting voltage waveforms after power limitation obtained at the active electrode and central points of the sensory epithelia of the posterior, anterior and saccular nerve with monopolar electrode configuration in the ampulla of the posterior SCC using the default values for the tissue properties

To present a more realistic electrode-tissue interface in the model, encapsulation resistivity was considered in addition to C_dl_ in the simulation. Simulation predictions with a monopolar electrode configuration, in which the active electrode was placed in the ampulla of the posterior SCC and a C_dl_ and scar tissue thickness of 500 µm was considered, showed that a voltage amplitude higher than 70 V was reached in the active electrode, which corresponds to point E in Fig. 2 (see Fig. 7a, solid black line). This voltage amplitude cannot be present at the active electrode when a power limitation of 5 mW is considered (see "Power Limitation"). Therefore, Fig. 7a also shows the current and the voltage waveforms at the electrode when the power limitation was considered (dashed lines). This leads to a considerable current reduction that could be applied through the active electrode: it decreases from 1 mA to 0.27 mA. Consequently, the voltage waveform was reduced to approximately 20 V. This value is above the compliance level that could be reached by a CI, which is ranging between 5 V and 7 V [38] leading to even lower current applied through the active electrode. The predicted voltage waveforms obtained by applying the power limitation are shown in Fig. 7b, where, during the phase gap between the cathodic and anodic phase, the plateau at 0 V was replaced with a smooth transition between the two phases. When comparing the predicted voltage waveforms obtained without considering the electrode-tissue interface (Fig. 6a), in the scenario in which the power limitation was applied, the voltage amplitude in the second scenario decreased by a quarter with respect to the voltage amplitude in the first scenario. Not only was the amplitude changed but also the voltage waveform shape: when the scar tissue and C_dl_ were included, the voltage during the two phases did not remain constant but instead decreased slowly before dropping down to 0 V at the end of the pulse.

### Neural Model

**Fig. 8:**
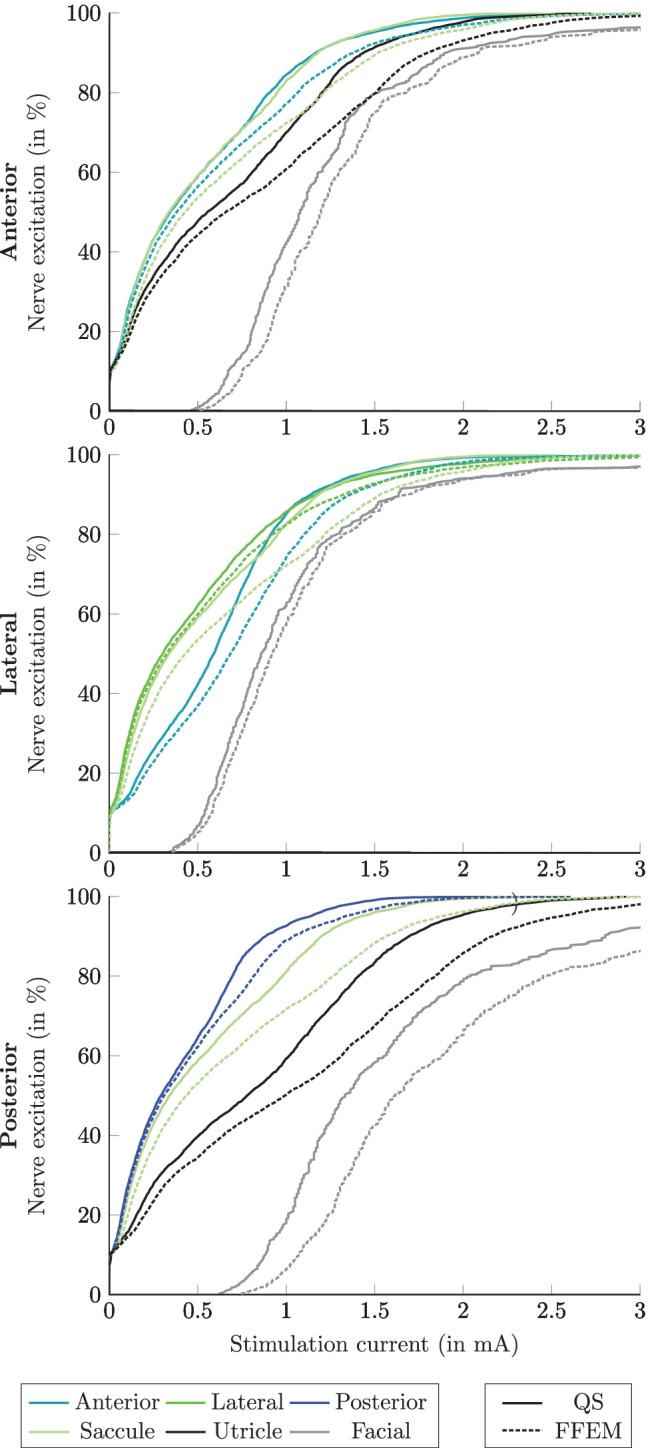
Simulated recruitment curves when applying the long stimulus waveform during monopolar electrode stimulation when the active electrode is located in the ampulla of the anterior SCC (top), the lateral SCC (center) and the posterior SCC (bottom). Nerve fiber recruitment curves obtained by performing the QS approach are shown with solid lines, while the recruitment curves obtained by the Fourier FEM without electrode-tissue interface are illustrated in dashed lines. The plots only show the corresponding target nerve, the two most activated non-target nerves and the facial nerve

**Fig. 9:**
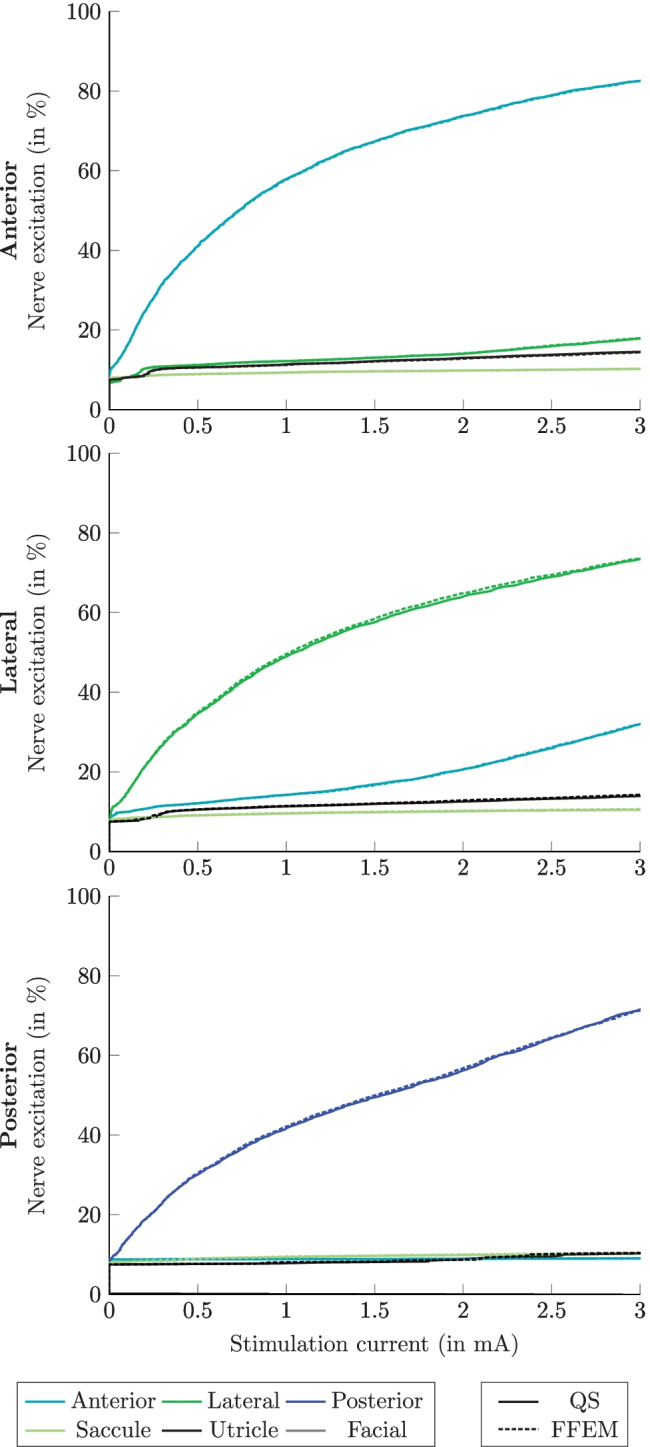
Simulated recruitment curves when applying the long stimulus waveform during bipolar electrode stimulation when the active electrode is located in the ampulla of the anterior SCC (top), the lateral SCC (center) and the posterior SCC (bottom). Nerve fiber recruitment curves obtained by performing the QS approach are shown with solid lines, while the recruitment curves obtained by the Fourier FEM without electrode-tissue interface are illustrated in dashed lines. The plots only show the corresponding target nerve and the three most activated non-target nerves (the facial nerve is not activated within this current stimulus amplitude)

The percentages of nerve fiber excitation related to the stimulation current amplitude for QS (obtained by considering only frequency-independent conductivities in the simulations for the different tissues in the model) and Fourier FEM, without electrode-tissue interface, during monopolar and bipolar transversal parallel electrode configurations, applying the long stimulus waveform, are shown in Figs. 8 and 9, respectively. In QS simulation predictions (solid lines) of monopolar stimulation of the posterior and lateral nerves, the stimulated recruitment curves show that the percentage of activated target nerve fibers is always higher than that of the other nerve structures at low currents, which is not the case for the anterior nerve, where both the anterior and saccular nerves are activated with the same stimulation current amplitude. In addition, in all monopolar stimulation scenarios, the saccular nerve shows the highest stimulation sensitivity of non-target nerves. For example, when the stimulation current is 0.5 mA, considering monopolar stimulation of the anterior nerve, the same excitation percentage (59 %) for the anterior and saccular nerve is obtained. Instead, considering the posterior nerve as the target nerve, the amount of excitation for the posterior nerve is 65 %, while for the saccular nerve, it is 58 %. Conversely, when simulating monopolar stimulation scenarios using the Fourier FEM (dashed lines in Fig. 8), the same behavior described previously in the predictions of the posterior and lateral target nerves was also present in the predictions of the anterior target nerve: the stimulation recruitment curves for anterior and saccular nerves did not overlap in this stimulation scenario. Moreover, by considering permittivity in the simulation, the nerve fiber recruitment curves are shifted higher in stimulus amplitude ranges, such that more current is required to activate the same number of nerve fibers. No differences in nerve fiber stimulation were found when comparing simulation predictions considering the C_dl_ to simulation predictions that neglected it (not shown).

**Fig. 10:**
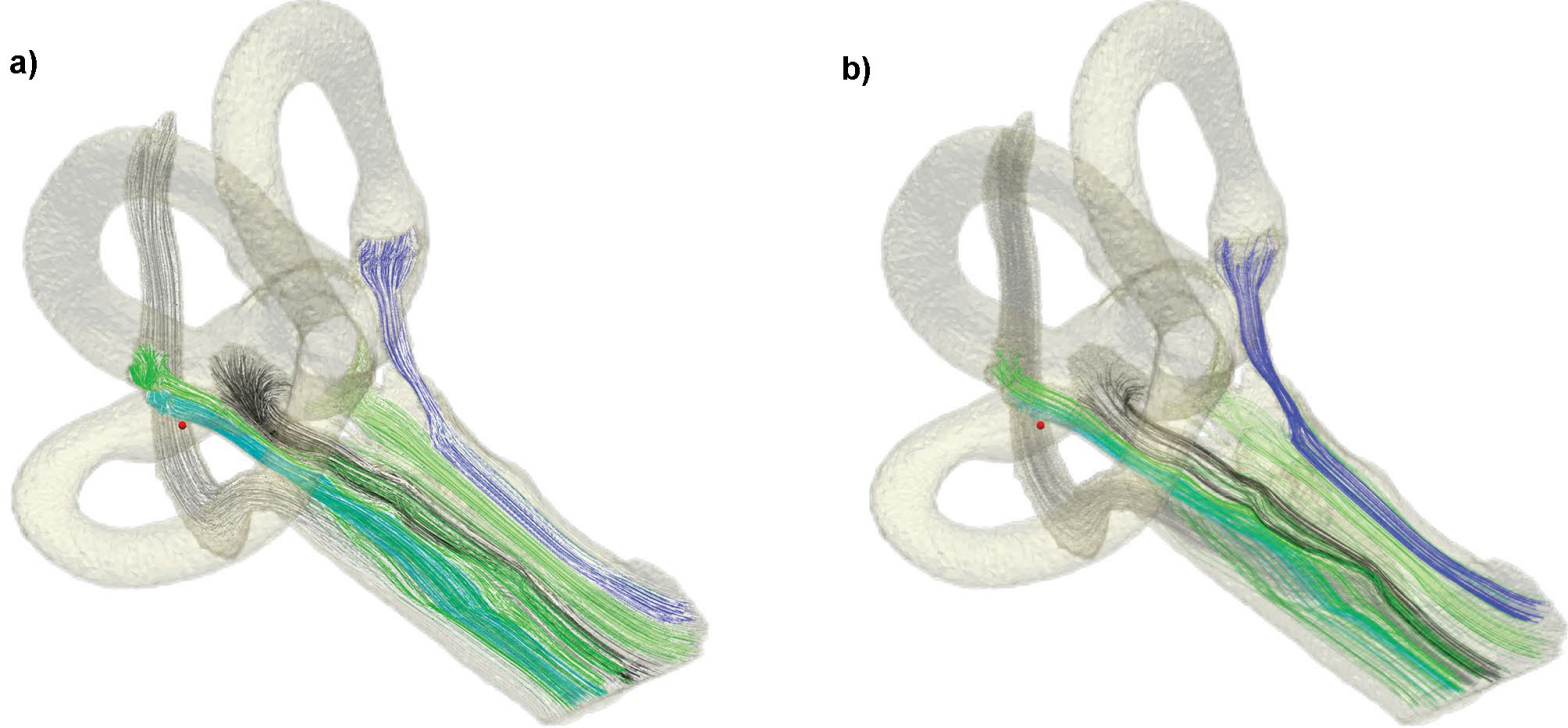
Nerve fiber pathways for each nerve: **a)** Nerve fibers activated during monopolar stimulation simulated using the Fourier FEM approach when the long stimulus waveform with an amplitude of 1 mA is applied at the active electrode (red sphere, located in the center of the ampulla of the anterior SCC); **b)** difference in active nerve fibers between QS and Fourier FEM when the same current amplitude and the same active electrode position is considered

Figure 10a shows the nerve fiber pathways and their activation when the long stimulus waveform with an amplitude of 1 mA is applied at the active electrode placed in the center of the ampulla of the anterior SCC (see Fig. 8 top) and the Fourier FEM simulation is considered. Moreover, at the same stimulus amplitude and active electrode position, the nerve fibers differences between QS and Fourier FEM are illustrated in Fig. 10b (for example, considering the excitation percentage of the anterior nerve for the QS approximation (84 %) and for the Fourier FEM (76 %) at 1 mA are subtracted, and the anterior nerve fibers difference between these percentage are colored in this figure; this approach is applied for each nerve separately).

Regarding bipolar electrode configuration, QS and Fourier FEM without electrode-tissue interface simulation produced analogous predictions: the target nerves demonstrated considerably higher sensitivity with respect to the non-target nerves. In addition, there was almost no difference between the simulated recruitment curves of the target nerves, and only small deviations were visible for the recruitment curves of non-target nerves. The percentage of nerve fiber excitation slightly increased when the C_dl_ was considered at both electrodes compared to the simulation scenarios neglecting C_dl_ described before (not shown).

**Fig. 11:**
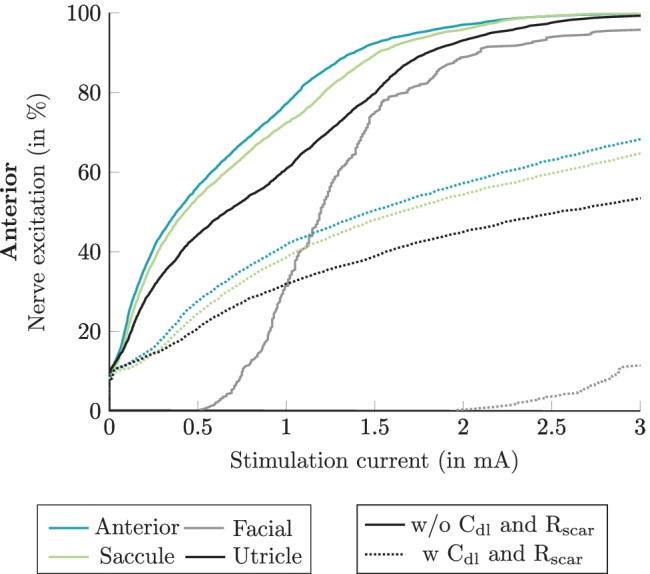
Simulated recruitment curves when applying the long stimulus waveform during monopolar electrode stimulation of the anterior nerve considering (dashed lines) and not considering (solid lines) polarization capacitance and a scar tissue of 500 µm thickness by performing the Fourier FEM simulation. The plot only shows the corresponding target nerve, the two most activated non-target nerves and the facial nerve

In Fig. 11, the simulated recruitment curves of the anterior monopolar stimulation scenario, applying the long stimulus waveform, using Fourier FEM with and without electrode-tissue interface (and scar tissue with a thickness of 500 µm and C_dl_) are shown. The effects of more realistic modeling of the electrode-tissue interface are visible for every considered nerve branch in the simulation. For example, considering a stimulation current of 0.5 mA, the percentage of the target nerve fiber excitation without considering C_dl_ and the scar tissue is 57 %, but when the scar tissue and polarization capacitance are considered, the percentage of active target nerve fibers is reduced to 27 %. In addition, at a stimulation current amplitude of 3 mA, all target nerve fibers are activated when no impedance of electrode polarization and scar tissue is considered. Only 70 % of the target nerve is activated when this additional impedance at the electrode surface is present.

**Fig. 12:**
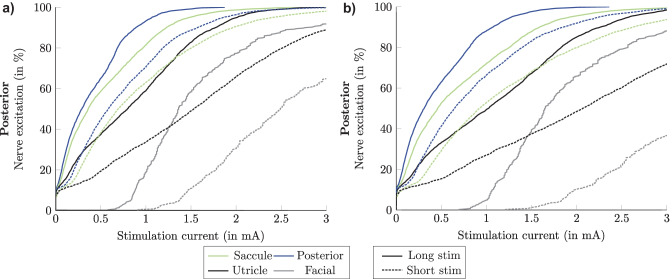
Simulated recruitment curves when applying long (solid lines) and short (dashed lines) stimulus waveforms during monopolar electrode stimulation of the posterior nerve performing: **a)** QS approximation and **b)** Fourier FEM simulations. The plot only shows the target nerve, the two most activated non-target nerves and the facial nerve

The comparison of the nerve fiber excitation curves between long and short current stimulus waveforms when performing both QS approximation and Fourier FEM simulations during monopolar electrode configuration is shown in Fig. 12. Both simulation scenarios using the short stimulus waveform produced a decrease of percentage of the nerve fiber excitation compared with the long stimulus waveform. This means that more current has to be applied to excite the same number of nerve fibers.

AUC values calculated for evaluating the selectivity of the QS approximation, the selectivity of the Fourier FEM considering and not considering the electrode-tissue interface as well as monopolar and bipolar electrode simulation scenarios are listed in Table 2: By using the Fourier FEM during monopolar electrode stimulation, the AUC values slightly increased compared with the QS approximation. Likewise, by comparing the long and short stimulus waveforms, for example when the active electrode is placed in the center of the ampulla of the posterior SSC, the AUC values change from 0.557 (long) and 0.553 (short) performing QS approximation to 0.585 (long) and 0.595 (short) applying the Fourier FEM. Furthermore, the AUC values obtained by performing bipolar electrode stimulations considering the electrode-tissue interface (only C_dl_ or both C_dl_ and R_scar_) during Fourier FEM did not produce a notable difference between all the ampullary nerves.

**Table 2:** AUC values obtained by applying the long stimulus waveform during monopolar and bipolar electrode configuration. When the electrode-tissue interface is considered (Fourier FEM with C_dl_ and R_scar_) the AUC values are calculated after applying the power limitation

Simulation	Electrode configuration	Anterior	Lateral	Posterior
QS	monopolar	0.4962	0.5198	0.5572
	bipolar	0.8521	0.7573	0.9009
Fourier FEM	monopolar	0.5250	0.5536	0.5852
	bipolar	0.8522	0.7574	0.9012
Fourier FEM with C_dl_	monopolar	0.5256	0.5486	0.5780
	bipolar	0.8523	0.7577	0.9013
Fourier FEM with C_dl_ and R_scar_ (500 µm)	monopolar	0.5243	0.5478	0.5776
	bipolar	0.8524	0.7578	0.9014

## Discussion

The aim of this study was to analyze the influence of the reactive component of impedance and the effects of the electrode-tissue interface of a simplified vestibular implant model in human vestibular anatomy on voltage waveforms and nerve fibers selectivity, two aspects not considered yet in vestibular implant simulations.

This goal was achieved by considering frequency-dependent permittivity values of the tissues in a Fourier FEM simulation environment as well as an instrumental electrode model applied to the electrodes of the implant to consider the polarization effects at the electrode-tissue interface. The AUC value was used as an indicator to quantify the nerve fibers selectivity.

Considering the reactive component of impedance in the simulation caused an effect on the morphology of chosen voltage waveforms in the model (as shown in Fig. 6) with respect to the QS approach: different amplitudes and slopes were obtained. These effects were visible in simulation predictions of monopolar electrode configurations for all three ampullary nerves (only the posterior monopolar scenario is shown). For the QS approximation, the time-dependent behavior of the potential distribution was obtained by first simulating the potential distribution caused by a unit current over the active electrode, which was then scaled by the time-dependent stimulus waveform. Therefore, the time-dependent voltage waveforms in the QS model correspond to scaled versions of the applied current stimulus waveform. In contrast, in the Fourier FEM, potential distributions for each frequency component of the current stimulus were obtained, and the phase shifts of the frequency components led to morphological changes of the predicted potential waveform at different locations in the model.

However, for bipolar transversal parallel simulation, the effects of the dielectric properties of the tissues were not as pronounced as in the monopolar configuration. In bipolar configurations, almost all the current was restricted between the active and sink electrodes (located close to each other), and only a small part of the current was spread over the other parts of the model. In addition, the permittivity value of the CSF, considered for the perilymphatic-endolymphatic space in which the electrodes were located, was three orders of magnitude smaller than that of bone and six orders of magnitude smaller than that of nerves considered in the model.

Conductivity and permittivity values were taken from Gabriel et al. [22]. The frequency-dependent values showed some uncertainty with higher ranges at lower frequencies. For this reason, a range boundary was defined for every considered dielectric property based on assumptions derived from literature (see "Tissue Properties"). A range of ±50 % was defined for the conductivity and permittivity of nerve tissue due to a lack of measurements available in literature, and since the variability of the conductivity of white matter, which shows comparable tissue properties, is around ±50 % [39]. However, the simulation predictions show that the most notable influence on the voltage waveform was observed when the tissue properties of the bone were considered at the upper and lower boundaries (see Fig. 6b and c). This can be explained by the fact that the bone is the component with the highest overall impedance between the active and reference electrodes in the model, using the monopolar electrode configuration.

This consideration is also shown on the RMSE values calculated for the same electrode configuration: when the tissue properties of the nerve and the CSF are considered at the lower and upper boundaries, RMSE values lower than 27 µV were obtained at the electrode and central point of the sensory epithelium of the posterior nerve, whereas RMSE values did not reach 8 µV at the other nerves. In contrast, the RMSE considerably increased when the lower and upper boundaries of the tissue properties of bone were considered.

The stimulus waveforms used during animal studies on chinchillas by Hayden et al. [12] had a phase duration from 100 to 230 µs with a phase gap from 0 to 50 µs and a current amplitude ranging from 0 to 2 mA. Hedjoudje et al. [13] considered a phase duration of 200 µs and a phase gap of 25 µs in their studies on rhesus monkeys with a current amplitude from 10 µA to 160 µA. On the other hand, Nguyen et al. [40] used two stimulus waveforms during measurements on patients: a longer waveform with a phase width of 200 µs and a phase gap of 50 µs, and a shorter waveform with a phase duration of 50 µs and a phase gap of 2.1 µs. Both stimulus waveforms had a current amplitude ranging from 100 µA to 950 µA. In this study, a long (phase duration: 200 µs, phase gap: 50 µs) and short (phase duration: 50 µs, phase gap: 2.1 µs) stimulus waveforms were considered same as in Nguyen et al. [40] and also comparable to the other listed studies. To the best knowledge of the authors, the effects at the electrode-tissue interface described in this paper have not been considered in simulations of vestibular nerve stimulation before. In addition, the power limitation aspect considered to properly evaluate these effects is based on a value obtained from the literature (P_MAX_ = 5 mW), and it might be different for a specific vestibular implant. The polarization capacitance and impedance of scar tissue layer around the electrodes are present in real life when a foreign structure is introduced into the human body. This extra tissue is characterized as a low conducting material, and it represents a barrier between the active electrode and the rest of the model. The scar tissue develops over time, and thus, the stimulation amplitudes required to stimulate the same amount of target nerve fibers will change over time.

The effects combining the polarization capacitance and the scar tissue around the active electrode during monopolar electrode configurations are clearly visible on the voltage waveform at the point inside the electrode (point E in the equivalent circuit of Fig. 2): when the long current stimulus appears (at 280 µs), the voltage increases immediately and continues to grow until the cathodic phase of the stimulus is finished (at 480 µs). Subsequently, it drops back to 0 V (see Fig. 7a). The same can also be seen during the anodic pulse phase. This progress was not visible on the active electrode and on the central points of the sensory epithelia of the posterior, anterior, and saccular nerves without considering the power limitation aspect. The reason is that, as mentioned above, the current applied through the electrode is not influenced by changes in impedance during the current-controlled stimulation. The effects of considering the power limitation, polarization capacitance, and impedance around the electrode are reflected in the voltage waveform (see Fig. 7b), and consequently, in the activation of the target nerve fibers (see Fig. 11).

The simulated recruitment curves obtained by performing the QS simulation in monopolar electrode configurations are comparable with the predictions obtained by Handler et al. [15] and Schier et al. [16]. The slight discrepancy between the simulation predictions is due to the different electrode sizes and the instrumental electrode model introduced in the simulation. Nevertheless, in monopolar configurations (see Fig. 8), taking the reactive component of impedance into account predicts a better selectivity for the target nerves with respect to the QS simulation (see Table 2, e.g., during posterior monopolar stimulation with the long stimulus waveform the AUC values are 0.5572 and 0.5852 for QS and Fourier FEM, respectively), due to the considered capacitive effects that delay changes in the extracellular potential and, consequently, decrease the nerve fiber activation percentage for the same stimulus current amplitude. In contrast, the same recruitment curves for all the ampullary nerves in both approaches when using bipolar simulation scenarios (see Fig. 9) must be associated with the restricted path of the current between the active and sink electrodes. However, considering the C_dl_ on both electrodes showed a slight increase in nerve fiber excitation (not shown) which does not considerably affect the AUC values compared with the AUC values obtained by performing the Fourier FEM simulation without considering the C_dl_ (see Table 2).

According to the simulations, during monopolar electrode configuration, the predictions of the Fourier FEM led to a slightly better selectivity for targeted nerve stimulation compared to the QS approach. For example, Table 2 shows that the AUC increases from 0.4962 to 0.5250 during anterior monopolar electrode configuration when the Fourier FEM is considered instead of the QS approximation. Like the predictions described by Hayden et al. [12], Handler et al. [15] and Schier et al. [16], a higher selectivity was obtained by bipolar stimulation (see Table 2) at the expense of higher stimulus currents required to activate the same percentage of targeted nerve fibers.

The nerve fiber activation obtained by applying the long stimulus waveform produced an increase of percentage of the nerve fiber excitation compared with the short stimulus waveform. This could be associated with the comparatively high amount of charge that is applied to the active electrode during the cathodic phase.

Our results indicate that the scar tissue around the electrodes has a considerable influence on the nerve fiber activation and therewith the vestibular stimulation (when considering the power limitation). This effect needs to be considered when configuring stimulation protocols in vestibular implant patients. On the other hand, the effects of the C_dl_ and therewith the electrode material appears to be negligible according to our simulations.

The model considered and simulations performed are subject to limitations: the surrounding components of the vestibular anatomy, such as dura mater with high resistivity in the medial direction, mastoid cells, and middle ear, were schematized by a bone sphere surrounded by a saline layer. Although a comparable simplification was considered in other works simulating vestibular implants [14–16], this might cause deviations in the voltage waveforms and in the simulated recruitment curves, especially during monopolar stimulation. In future work we consider using more realistic anatomical surrounding in the simulation environment by embedding the inner ear geometry into a realistic human head model (e.g., by integrating the high-resoluted 3D model in the publicly available MIDA head model [41]). Furthermore, an explicit reference electrode should be introduced in the model (e.g., on the implant housing) instead of considering the reference electrode (potential equal to 0 V) as the outer boundary of the saline layer for monopolar and bipolar simulation scenarios. Both aspects will influence current flow in the model, potentially leading to deviations in voltage waveforms and nerve fiber activation. However, it is assumed that the predicted effects will not be considerable for bipolar electrode configurations, since the current is mostly limited between the electrodes that are placed close to each other (i.e., active and sink electrodes).

The dielectric tissue properties and corresponding range boundaries used in the simulations were based on literature with some uncertainty (mainly at low frequencies). The conductivity and permittivity of the different structures considered in the 3D model produced changes in the predicted voltage waveforms and nerve fiber activation when comparing the QS approximation to the Fourier FEM approach. Particularly, it has been shown that the tissue properties of the bone must be evaluated correctly, because the change of these properties showed a strong influence on the voltage waveforms during monopolar simulation scenarios.

Furthermore, the value of the C_dl_ used in the simulations is an average value for metal in an aqueous solution. For this reason, an evaluation of this polarization capacitance should be considered for electrodes used in the models to obtain a more representative value. Moreover, the maximum power used for electrical stimulation (5 mW) was based on cochlear implants, and it limits the current density applied through the active electrode. This makes it possible to properly simulate the effects of the electrode-tissue interface on the predicted voltage waveforms and, consequently, on the nerve fiber activation.

The presented simulation methodology allows for considering the dielectric properties of the tissue during simulation of the human vestibular system stimulation. Although it has been argued by other authors that the application of the QS approaches would be justified [12, 13, 17], justifying the application of QS approaches, it has been shown in Table 1 and in Pleshkov et al. [23] that the permittivity of the tissues should be considered. For this reason, the Fourier FEM provides notable information about the possible behavior of the electrically stimulated vestibular system. Therefore, it can be concluded that the effect of tissue permittivity and the instrumental electrode model including polarization capacitance and scar tissue, should be considered to improve the credibility of the simulations.

## Supplementary Information

Below is the link to the electronic supplementary material.

Supplementary file1 (PDF 130 KB)
