# Supplementary material for: Computer Simulation of the Electrical Stimulation of the Human Vestibular System: Effects of the Reactive Component of Impedance on Voltage Waveform and Nerve Selectivity
Source: J Assoc Res Otolaryngol. 2022 Sep 1;23(6):815–33. doi: 10.1007/s10162-022-00868-w (PMC9789245; doi:10.1007/s10162-022-00868-w)
Supplement: Supplementary file 1 — Supplementary file1 (PDF 130 KB) [file 10162_2022_868_MOESM1_ESM.pdf]

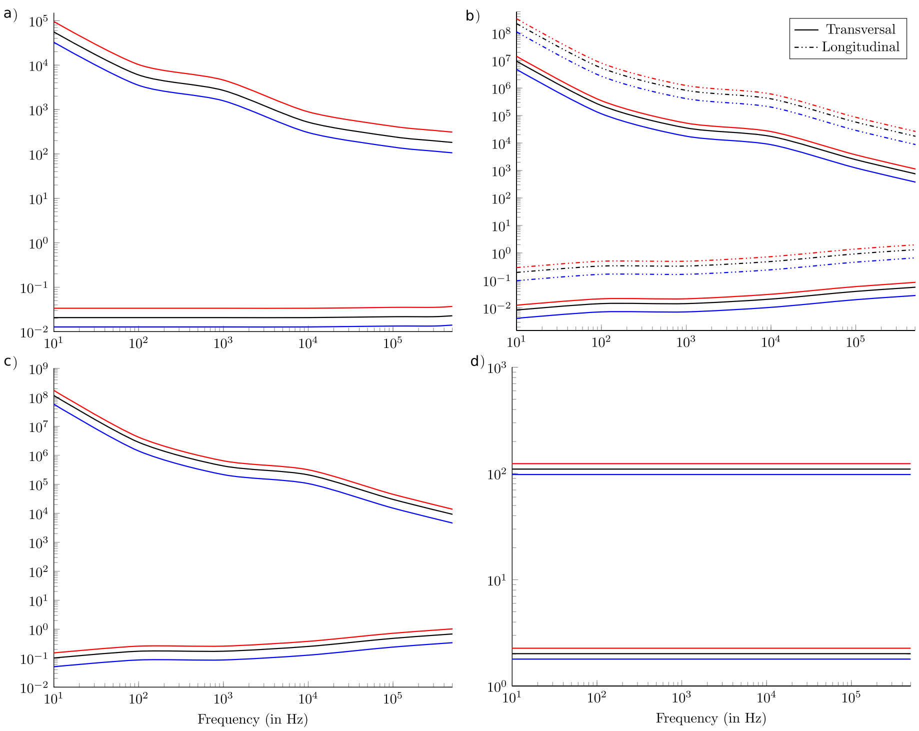

Figure 1: Tissue properties used during the simulation for the different tissue structures considered: a) Bone, b) Nerve, c) Cochlear nerve and d) CSF. The conductivity is indicated on the lower part of the plots and is given in $\mathrm{S\,m^{-1}}$, while the relative permittivity is shown on the upper part of the plots. The default value ($\gamma(\mathrm{f})$) is drawn in black, the lower boundary ($\gamma^{\downarrow}(\mathrm{f})$) in blue and the upper boundary ($\gamma^{\uparrow}(\mathrm{f})$) in red.

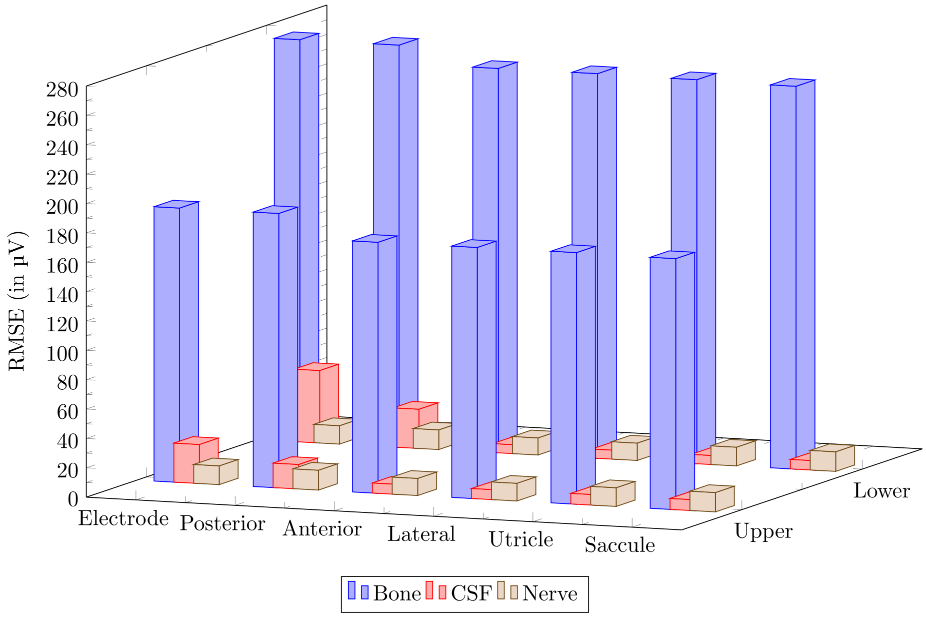

Figure 2: RMSE obtained at the active electrode and central points of the sensory epithelia of the posterior, anterior, lateral, utricular and saccular nerve, which varying tissue properties, by performing the Fourier FEM with monopolar electrode configuration when the active electrode is positioned in the center of the ampulla of the posterior SCC and the long stimulus waveform is applied. In the front row the RMSE values between the default value and upper range boundary are shown, while in the back row the RMSE is calculated between the default value and the lower range boundary.
